# Plastic leachates promote marine protozoan growth

**DOI:** 10.1093/ismejo/wraf195

**Published:** 2025-08-28

**Authors:** Jessy Le Du-Carrée, Cristina Romera-Castillo, Rodrigo Almeda

**Affiliations:** University Institute for Research in Sustainable Aquaculture and Marine Ecosystems (ECOAQUA), University of Las Palmas de Gran Canaria, Campus Universitario de Tafira, E-35017 Canary Islands, Spain; CSIC, Institute of Marine Sciences, Passeig Marítim de la Barceloneta 37–49, E-08003 Catalonia, Spain; University Institute for Research in Sustainable Aquaculture and Marine Ecosystems (ECOAQUA), University of Las Palmas de Gran Canaria, Campus Universitario de Tafira, E-35017 Canary Islands, Spain

**Keywords:** microplastics, leachate, marine protozoans, heterotrophic dinoflagellate, DOC, osmotrophy

## Abstract

Millions of tons of plastic enter the ocean annually, yet the effects of their leachates on the microbial loop are poorly understood. This study investigates how dissolved organic carbon released from field-collected plastics and a bioplastic influences the growth of the protozoan *Oxyrrhis marina* and its associated bacterial community. Plastics increased dissolved organic carbon concentrations in seawater by 5 to 34-fold, stimulating *O. marina* growth by up to an order of magnitude compared with the control. After exposure to conventional beach plastic leachates and bioplastic leachates, *O. marina* exhibited growth rates up to 0.3 and 0.4 d$^{-1}$, respectively, even in the absence of microalgal prey. We estimated that each gram of microplastics could lead to daily assimilation of up to 0.7 g of carbon per gram of protozoan, indicating that plastic-derived carbon enhances heterotrophic metabolism in the microbial loop through osmotrophy. Given that autotrophic prokaryotes are negatively impacted by plastic leachates and that plastic pollution is expected to triple in the coming decades, plastic leaching could alter the balance between microbial primary production and heterotrophy in the ocean.

## 1 Introduction

Microplastic pollution in aquatic ecosystems has emerged as a major environmental concern over the last decade [1–3]. Millions of tons of plastics enter the ocean every year [4, 5], and plastic litter in aquatic ecosystems is projected to triple by 2040 [6]. Recent research on plastic pollution has revealed that complex mixtures of plastic additives are released into the surrounding water through leaching [7]. Ecotoxicological studies of these plastic-leached compounds indicate their significant contribution to plastic toxicity [8], with potentially harmful effects on aquatic organisms [9–11]. For instance, plastic leachates most commonly exert neutral or adverse effects on planktonic organisms [12]. Conversely, the growth of marine heterotrophic bacteria is stimulated by the dissolved organic carbon (DOC) released in plastic leachates, which they utilize as a nutrient source [13–15]. This response in heterotrophic bacterioplankton is further enhanced by weathering processes that affect plastics [16]. However, positive and hormetic effects of plastic leachates on eukaryotic plankton, especially those within the microbial loop, remain insufficiently explored despite their implications for the functioning and dynamics of marine food webs.

Heterotrophic dinoflagellates are ubiquitous and abundant protists within planktonic communities but are understudied compared with other marine organisms [17]. Heterotrophic dinoflagellates are important predators of bacteria, heterotrophic nanoflagellates, and phytoplankton, and they serve as prey for a wide range of organisms, from other protists to metazoans. As significant components of the microbial loop in marine systems [18], heterotrophic dinoflagellates play a crucial role in the trophic pathway where bacterioplankton convert dissolved organic matter into biomass, consumed by heterotrophic protists. This process connects the microbial loop to the classical grazing food chain. Although several studies have confirmed the ingestion of microplastics by heterotrophic dinoflagellates, our knowledge of the effects of plastic leachates on these important planktonic organisms remains limited.

Assessing the impact of plastic leachates and their transformation products on the marine food web is challenging due to the diverse and complex chemical formulations of plastics composed of polymers, various synthetic additives, and manufacturing byproducts. Additionally, microplastics in aquatic systems also absorb and accumulate a mix of hydrophobic chemicals already present in the environment. Most current knowledge on the effects of plastic additives on plankton comes from studies on virgin/unweathered microplastics [19]. The influence of aging and weathering on plastic leaching remains poorly understood, highlighting the need for bioassays with field-collected plastics. Another research gap in plastic pollution is understanding the ecological effects of plant-based materials—”bioplastics (BioP)”—marketed as environmentally friendlier alternatives to traditional plastics. Several studies have reported adverse effects of certain BioP on marine organisms [20, 21], with impacts that are comparable with, or even exceed, those of conventional plastics [22]. BioP and their leachates may affect microbial communities in two opposing ways: by supplying organic carbon that can stimulate microbial growth, and by introducing toxic compounds that may inhibit it. This dual potential impact of BioP remains poorly understood, underscoring the need for more comprehensive research on their effects on marine microbial communities and the microbial loop [23].

The microbial loop encompasses planktonic bacteria, viruses, and protozoans [18] and plays a major role in oceanic carbon cycling [24]. It provides key pathways for the incorporation of DOC into the marine food web, facilitating the transfer of carbon to higher trophic levels and thereby contributing to the recycling of carbon within the classical food web [24]. This process also enhances carbon sequestration through the biological carbon pump [25, 26]. Emerging research suggests that the microbial loop may act not only as a link for carbon recycling but also as a carbon sink by transforming labile DOC into more recalcitrant forms, such as ”refractory” DOC, which are resistant to microbial degradation and can persist in the ocean for extended periods [27–30]. This insight has given rise to the concept of the Microbial Carbon Pump [29], which highlights the role of microbial processes in long-term oceanic carbon storage and global warming mitigation. Small planktonic protozoans, which consume bacteria and serve as prey for metazooplankton, contribute to both dissolved and particulate carbon pools, acting as a crucial link between the microbial loop and the classical marine food web [25]. Additionally, some protozoans influence DOC dynamics through osmotrophy, the absorption of dissolved organic matter, highlighting their multifaceted role in microbial carbon cycling [31, 32]. In the context of rising anthropogenic CO$_{2}$ emissions, global warming, and escalating plastic pollution, understanding how the microbial loop responds to plastic-derived leachates is increasingly critical. Its capacity to assimilate plastic-derived DOC through various microbial pathways may have significant implications for both carbon cycling and the mitigation of pollution in marine ecosystems.

In this study, we aim to assess the response of marine planktonic protozoans to leachates from field-collected conventional plastics and virgin BioP. *Oxyrrhis marina*, a small (ca. 20$\;\mu $m) heterotrophic dinoflagellate with a global distribution in coastal waters [33], was used as a model marine protozoan. *O. marina* is recognized as a model organism in various disciplines such as ecology and evolution [34] and has been utilized in several toxicological studies [35, 36]. Although several authors have demonstrated that *O. marina* can ingest microplastics [35, 37, 38], the effects of plastic leachates on *O. marina* or any other heterotrophic dinoflagellates remain unexplored. Heterotrophic nutrition strategies in marine dinoflagellates are diverse and include osmotrophy, the uptake of dissolved organic molecules, as a potential pathway for carbon assimilation [31, 39, 40]. Based on this, we hypothesized that plastic-derived DOC could stimulate *O. marina* growth either by directly supplying bioavailable carbon substrates or indirectly by enhancing its associated bacterial communities. In this study, we investigated how plastic-leached DOC affects the growth of *O. marina* and the abundance of its associated bacteria. We estimate how much plastic-derived carbon can be assimilated by *O. marina* and discuss the broader implications for the marine food web.

## 2 Materials and methods

**Figure 1:**
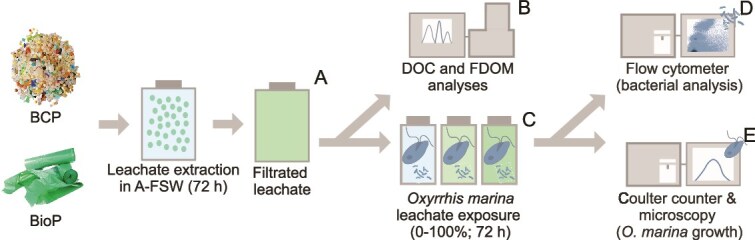
Graphical representation of the experimental design showing (A) leachates prepared from BCP and new BioP in A-FSW, which also served as the control; (B) measurement of DOC and FDOM; (C) incubation of the leachates with *O. marina* for 72 h; (D) analyze of bacterial abundances; (E) quantification of *O. marina* concentrations.

The experimental design and methodological and analytical procedures are presented in detail in the following sections (For a graphical overview see Fig. 1).

### 2.1 Plastic types and obtaining of microplastics

**Figure 2:**
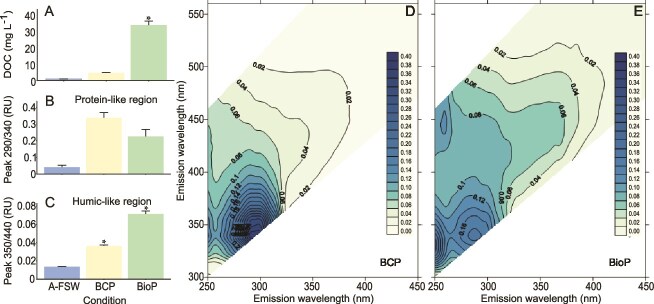
Characteristics of DOC and FDOM in leachates from micronized BCP and BioP incubated with A-FSW for 72 h. A: concentration of DOC (mg ml$^{-1}$) in leachates from A-FSW (control), BCP, and BioP after 72 h of incubation, prior to *O. marina* inoculation. B: maximum peak fluorescence values (R.U.) in the protein-like region and (C) the humic-like region for A-FSW, BCP, and BioP leachates. D and E: net Excitation Emission Matrices of leachates from BCP and BioP, with the control, A-FSW, subtracted (mean, $\mathit{n} = 3$). Significant differences from the control means are marked with “*” ($\mathit{P} < 0.05$, $\mathit{n} = 3$). Data are presented as mean $\pm $ standard error. Measurements were conducted during the second *O. marina* growth experiment.

To obtain conventional microplastics from environmental samples, field macroplastics, mainly consisting of bottles, caps, hard containers, bags, and ropes, were collected from three different beaches on the French Atlantic coast. These items were cut into 5 cm squares and ground using an ultracentrifuge mill (ZM200, Retsch) with dry ice. The composition of the microplastic mixture by dry weight was 46% polyethylene (PE), 40% polystyrene (PS), 12% polypropylene (PP), and 2% polyethylene terephthalate (PET). More details on the collecting locations and procedures can be found in another article [41]. A commercial home-compostable bag with starch (Mater-bi) composed of polybutylene terephthalate (supplier: BioBag, USA) was used as BioP. Mater-Bi is known to be composed of $\sim $70% PBAT, 20% starch, and 10% additives [42]. The bags were also cut into 5 cm squares and micronized using an ultracentrifuge mill to obtain microplastics. Each type of ground plastic was dry-sieved through an ISO-certified stainless-steel sieve to obtain microplastics $\leq 250\ \mu $m [41]. The particle size distribution between 0.4 and 2000 $\mu $m for each type of plastic (fraction $\leq 250\ \mu $m) was measured using a laser diffraction particle analyzer (LS I3 320, Beckman Coulter). The data are available in Fig. 2 of a previously published study [41].

### 2.2 Leachates preparation

The seawater used throughout the study was autoclaved filtered seawater (A-FSW) of oceanic origin. It was collected nearshore ($27^\circ 59\prime 27^{\prime \prime }\mathrm{N},\ 15^\circ 22\prime 01^{\prime \prime }\mathrm{W}$ ), filtered through a 5$\ \mu $m filter, sterilized using a UV lamp, and stored in a 7} m-deep well at the Spanish Bank of Algae installations in Gran Canaria. Before preparing the leachates for the bioassays, the seawater underwent triple filtration: first with an activated carbon coconut shell filter (5$\ \mu $m), followed by a polypropylene melt-blown filter (1$\ \mu $m), and finally a polyethersulfone membrane filter (0.1$\ \mu $m). The water was then autoclaved at 2 atm and 120$^{\circ }$C for 20 min.

Leachate from the different plastic types was prepared following a classic four-step procedure [41]. Microplastics ($\leq 250\ \mu $m) were incubated at 20$^{\circ }$C in darkness and for 72 h in glass bottles rotating at 15 rpm using rollers. The concentration of microplastics was 1 g l$^{-1}$. After incubation, samples were filtered to remove microparticles using Whatman glass microfiber filters with a pore size of 0.7 $\mu $m and a glass vacuum filtration system. During the second experiment involving *O. marina* exposure, at the end of the leaching experiment, the filtered leachates and A-FSW (used as a control) were collected and stored at -20$^{\circ }$C in acid-washed vials for DOC and fluorescent dissolved organic matter (FDOM) analyses.

In this article, the term ”leachate dilution” is used to quantify exposure, where 100% corresponds to an exposure of 1 g l$^{-1}$ of plastic equivalent, meaning leachates obtained from 1 g of plastic particles in 1 l of water. A 0% exposure represents the A-FSW (control seawater) with 0 g l$^{-1}$ of plastic.

### 2.3 *O. marina* culture

The strain of *O. marina* used in this study (OXY-BCN) was originally isolated off the coast of Barcelona in 1996 by A. Calbet. Experiments were conducted using non-axenic monocultures of *O. marina* maintained in glass bottles with A-FSW at a salinity of 35‰ and a temperature of 20$^{\circ }$C in the dark. The *O. marina* cultures were fed *ad libitum* three times a week with the cryptophyte *Rhodomonas salina*. *R. salina* culture was grown in B1 media (salinity=35‰) and kept at 20$^{\circ }$C with an illuminance of 80 $\mu $mol m$^{-2}$ s$^{-1}$ and a 16:8 h day: night photoperiod. Before initiating a bioassay, the *O. marina* cultures were starved for 72 h, and then the culture was observed under the microscope to ensure that *R. salina* cells did not remain in the culture or inside *O. marina* cells.

### 2.4 Bioassays

We conducted two bioassays with *O. marina* and two types of plastic leachates to assess the impact of these leachates on the population growth of this species. In the first experiment, we used 34 ml acid-washed glass bottles. Each bottle was filled with 33 ml of exposure media and 1 ml of *O. marina* culture (13600 cells/ml), resulting in an initial cell concentration of 400 cells/ml. Leachate dilutions of 100%, 50%, 25%, 12.5%, and 6.25% were tested in triplicate. The bottles were incubated in the dark at 20$^{\circ }$C with constant shaking at 15 rpm for 72 h. In the second experiment, the exposure was conducted in 50 ml glass bottles containing 23 ml of test media. Each bottle was inoculated with 2 ml of *O. marina* culture at 25 000 cells/ml, yielding an initial concentration of 2000 cells/ml. In this case, leachate dilutions of 100%, 33%, 10%, and 3.3% were used in triplicate. The exposure time and incubation conditions were identical to those employed in the first experiment. At the end of both experiments, samples were collected to assess changes in cell concentration. For the first experiment, the sample was transferred to tubes and fixed with 1% Lugol’s solution for later microscopic analysis. In the second experiment, each bottle was carefully mixed, and 2 ml of the mixture was preserved in cryovials with 1% glutaraldehyde, maintained in the dark for 10 min before being stored SI t −80$^{\circ }$C until analysis. The remaining sample was used to analyze *O. marina* cell concentration and size.

### 2.5 Sample analyses and calculations

#### 2.5.1 *O. marina*

In the first experiment, the counting was done using a Leica inverted microscope (DMi1) and a cell counting chamber (1 ml Sedgewick rafter cell) using samples fixed with a Lugol’s solution (1% final concentration). In the second experiment, the count and size of cells were determined by utilizing a Coulter Multisizer 4e (Beckman Coulter). Two measures per sample were conducted. The mean equivalent spherical diameter ($\ \mu $m) of the cells for each replicate was noted (Table S1, Supplementary Information).

The specific growth rate (GR) of *O. marina* was calculated as:

To estimate the growth of *O. marina* in terms of carbon, its carbon content was calculated using a C:vol conversion factor [43]: Cell carbon content (in pg C cells$^{-1}$) = 0.123 * volume (in $\mu \text{m}^{3}$). 


(1) \begin{align*}& \text{GR} = \frac{\ln(C_{f}) - \ln(C_{i})}{t}\end{align*}

Equation 1. Where $C_{f}$ and $C_{i}$ are the cell concentrations (cells ml$^{-1}$) at the final and initial incubation times, respectively, and $t$ is the exposure time in days. 


(2) \begin{align*}& C_{\text{respiration}} = R \times C_{\text{mean}} \times t\end{align*}

Equation 2. Where $R$ is the daily C-specific respiration rate (the value of 0.355 d$^{-1}$ was obtained from the average values of previous studies, assuming a Q10 of 2 [44, 45]), $C_{\text{mean}}$ is the average carbon biomass of *O. marina* in $\mu $g C ml$^{-1}$ during the incubation, and $t$ is the incubation time in days. 


(3) \begin{align*}& C_{\text{assimilation}} = \frac{C_{\text{growth}} + C_{\text{respiration}}}{C_{\text{mean}}} \times \frac{1}{t}\end{align*}

Equation 3. Where $C_{\text{growth}}$ is the increase in carbon biomass in the population after 72 h of incubation in $\mu $g C ml$^{-1}$, $C_{\text{respiration}}$ is the total carbon respiratory losses ($\mu $g C ml$^{-1}$), $C_{\text{mean}}$ is the average carbon biomass of *O. marina* in $\mu $g C ml$^{-1}$ during the incubation, and $t$ is the incubation time in days. 


(4) \begin{align*}& \text{PCU} = \frac{C_{\text{growth}} + C_{\text{respiration}}}{\text{[DOC]}} \times 100\end{align*}

Equation 4. Where $C_{\text{growth}}$ is the increase in carbon biomass in the population after 72h of incubation in $\mu $g C ml$^{-1}$, $C_{\text{respiration}}$ is the total carbon respiratory losses ($\mu $g C ml$^{-1}$), and [DOC] is the initial concentration of the DOC in $\mu $g ml$^{-1}$


(5) \begin{align*}& I_{\text{max}} = \frac{71.3 \times 20 \times 10^{-9} \times C_{\text{avg}} \times 24}{C_{\text{mean}}}\end{align*}

Equation 5. Where 71.3 is the maximum ingestion rate of *O. marina* on bacteria (bacteria ingested per *O. marina* cell per hour) at satiating levels, according to a previously published method [46], 20 fg is the average carbon content per bacterial cell [47], $C_{\text{avg}}$ is the mean cell concentration, between $C_{f}$ and $C_{i}$, of *O. marina* in cells ml$^{-1}$ over 72 h of incubation, $C_{\text{mean}}$ is the average carbon biomass of *O. marina* in $\mu $g C ml$^{-1}$ during the incubation, and $t$ is the incubation time in days. The calculated $I_{\text{max}}$ values were further adjusted to the experimental temperature using a Q_10_ of 2.

The carbon respiratory losses ($C_{\text{respiration}}, \mu{\text{g}}\,\textup{C}\,\text{ml}^{-1}$) during the incubation were estimated as:

The Carbon assimilation of *O. marina* ($C_{\text{assimilation}}, \mu \,\text{g}\ {\textup{C}_{O.\ marina}}^{-1}$) for the total incubation time (72 h) was calculated as:

The Percentage of Carbon Utilization (PCU, %) was calculated as:

Based on the functional response of *O. marina* feeding on bacteria reported by a previously published study [46], and taking into account the bacterial concentrations observed in our experiment ($>10^{6}\,\text{cells}\,\text{ml}^{-1}$), the maximum daily carbon-specific ingestion rate of *O. marina* ($I_{\text{max}}$, in $\mu $g C $\mu $g C$^{-1}$ d$^{-1}$) feeding on bacteria was calculated as follows.

#### 2.5.2 Flow cytometry analysis

Bacterial abundance was measured by flow cytometry (Cytoflex S, Beckman Coulter) [48]. Briefly, the bacterial PCU has been calculated following the equation 2 described above, considering a bacterial carbon content of 20 fg C cells$^{-1}$ [49]. As bacterial growth was found in the control, the DOC present in the A-FSW was considered.

#### 2.5.3 DOC analysis

DOC was measured in the A-FSW used as control and in the two types of leachates (BioP and BCP, at 1 g l$^{-1}$) during the second experiment, using a Shimadzu TOC-V organic carbon analyzer after CO$_{2}$ was removed by vigorous sparging with high-purity N$_{2}$. The instrument performance was tested daily with the DOC reference materials provided by D.A. Hansell (University of Miami). We obtained average concentrations of 45.39 $\mu $mol l$^{-1}$ for the deep ocean reference (Batch 23–Lot 02–23) minus blank reference materials. The nominal DOC values provided by the reference laboratory was 45.3 to 46.9 $\mu $mol l$^{-1}$.

#### 2.5.4 Fluorescent dissolved organic matter

FDOM samples were measured using a JASCO FP-8350 spectrofluorometer in a 1 cm quartz cell. Excitation-Emission matrices were obtained for emission wavelengths from 300 to 560 nm (2 nm steps) and excitation wavelengths from 240 to 450 nm (5 nm steps) with a bandwidth of 5 nm and an integration time of 0.1 s. Raman peak at 350 nm, using Milli-Q water, was measured daily for sample blank correction. The following three standards were run daily: (1) a sealed Milli-Q cuvette (Perkin Elmer) scanned between 365 and 450 nm exciting at 350 nm; (2) a P-terphenyl block (Stranna) that fluoresces in the amino acid region, between 310 and 600 nm, exciting at 295 nm; and (3) a Tetraphenyl butadiene block (Stranna) that fluoresces in the humic region, between 365 and 600 nm, exciting at 348 nm. The drEEM 0.2.0 toolbox was used to standardize the Excitation-Emission matrices [50]. Inner filter effect was addressed by correcting Excitation-Emission matrices using the absorbance-based approach, and data were standardized and normalized to Raman Units (RU) based on measurements of the Raman peak at 350 nm. Absorbance spectra used for the corrections were performed using a JASCO V-750ST UV/Vis spectrophotometer.

### 2.6 Data processing and statistical analyses

Statistical analyses and data processing were conducted using R software [51]. Visualization of the results was carried out with the ggplot2 package [52]. The normality of quantitative datasets was assessed using the Shapiro–Wilk test. In contrast, homoscedasticity was evaluated with Levene’s test for parametric data and the Fligner–Killeen test for nonparametric data. For datasets meeting the assumptions of normality and homoscedasticity, one-way analysis of variance (ANOVA) was applied for mean comparisons, followed by Dunnett’s post hoc test [53]. If the data were normal but heteroscedastic, Welch’s one-way ANOVA was utilized [54], followed by the Tamhane–Dunnett post hoc test [55]. For non-normal data, the Kruskal–Wallis test was used for mean comparison, with Dunn’s post hoc test for pairwise comparisons [56]. A significance threshold of $\mathit{P} < 0.05$ was applied across all tests. Correlation analyses between variables were performed using the lmrob function from the robustbase package [57]. To validate the concentration-dependent effect statistically, a two-parameter log-logistic (LL.2) model was fitted to the growth curves using the drc package. The lack-of-fit test was used to compare the model with a simpler group-wise (ANOVA) model. Principal component analysis (PCA) was conducted using the FactoMineR [58] and factoextra [59] packages to explore the data structure and identify key contributing variables.

## 3 Results and discussion

Our overarching hypothesis was confirmed because exposure to leachates from micronized beach conventional plastics (BCP) and BioP enhanced the growth of heterotrophic protozoan *O. marina* in the absence of phytoplankton prey. The significant effects observed, with BCP doubling and BioP tripling protozoan growth, indicate that plastic-derived DOC is incorporated into the microbial loop not only by hetetrophic bacteria [13, 14, 60] but also by protozoans. The stimulation of the marine microbial loop by plastic pollution may therefore impact the dynamics of the marine foob web.

### 3.1 DOC and FDOM in plastic leachates

After 72 h of incubation, the DOC concentrations in the leachates from the BCP and the BioP increased by $\sim $5 to 34 times, respectively, compared with the control (Fig. 2A). The DOC concentration in the control was around 1 mg l$^{-1}$ and only the BioP treatment showed a statistically significant increase compared with the control ($\mathit{P} = 2.73e-2$, $\mathrm{df} = 2$, $\chi ^{2} = 7.2$; Fig. 2A). This substantial release of DOC into seawater was previously reported in another study [61]. In addition, a previous study showed that BioP such as PLA (polylactic acid, another biodegradable biobased polymer) released DOC levels similar to petrochemical-based materials [61], the studied BioP Mater-Bi released six times more carbon than the weathered conventional particles. The specific composition of BioP, primarily derived from starch, associated with its micronization might explain this difference. For weathered particles, although some soluble plastic compounds may have been lost during environmental exposure, micronization and aging can enhance the release of DOC from conventional plastics, highlighting a potentially greater impact on microbial communities [16].

The FDOM excitation-emission matrices of BCP and BioP leachates (Fig. 2D and E) revealed a predominant peak in the fluorescent protein-like region, with a maximum around Ex/Em 290 nm/340 nm. This peak coincides with peak-T commonly found in natural DOM but with the excitation lightly shifted to longer wavelengths [62] and has also been described for other plastic types [16, 61, 63]. The intensity of this peak tended to be higher for the plastic leachates than for the control. BCP leachates (0.34 R.U.) presented a higher fluorescence intensity than BioP (0.23 R.U.) but the difference was not significant (Fig. 2B). Both plastic leachates also present a fluorescent peak at Ex <250 nm between 300 and 385 nm of emission. The BioP leachates showed an additional peak at Ex/Em 250 nm/447 nm, which is not present in the BCP leachates. Additionally, both leachates exhibited fluorescence signal in the humic-like region, with higher values for BioP, which presented a maximum at around Ex/Em 350 nm/440 nm coinciding with peak-C commonly found in natural DOM. Both plastic leachates presented significantly higher intensity at Ex/Em 350 nm/440 nm than the control ($\mathit{P} = 4 \times 10^{-4}$, $\mathrm{df} = 2$, $\mathit{F} = 316.0$) and the values of BioP (0.71 R.U.) were also significantly higher than the leachates of BPC (0.36 R.U., Fig. 2C). The main protein-like peak found here was close to peak-T, associated with protein, amino acids, and polyaromatic hydrocarbons (PAHs). In this study, the fluorescence likely corresponds to compounds such as additives or impurities rather than proteins or amino acids [16]. Substances fluorescing in that region were labile to microbial uptake in previous studies with plastic leachates [16, 61]. This study also showed that PLA did not release FDOM in the protein-like region, and the release of DOC was similar to that of the petro-based LDPE plastic, suggesting that Mater-Bi used here is probably degraded faster than conventional plastic, releasing a higher amount of DOC. Differences in chemical composition among plastics also lead to different optical signatures of the leached material.

### 3.2 Marine protozoan growth stimulation by plastic leachates: trophic pathways

**Figure 3:**
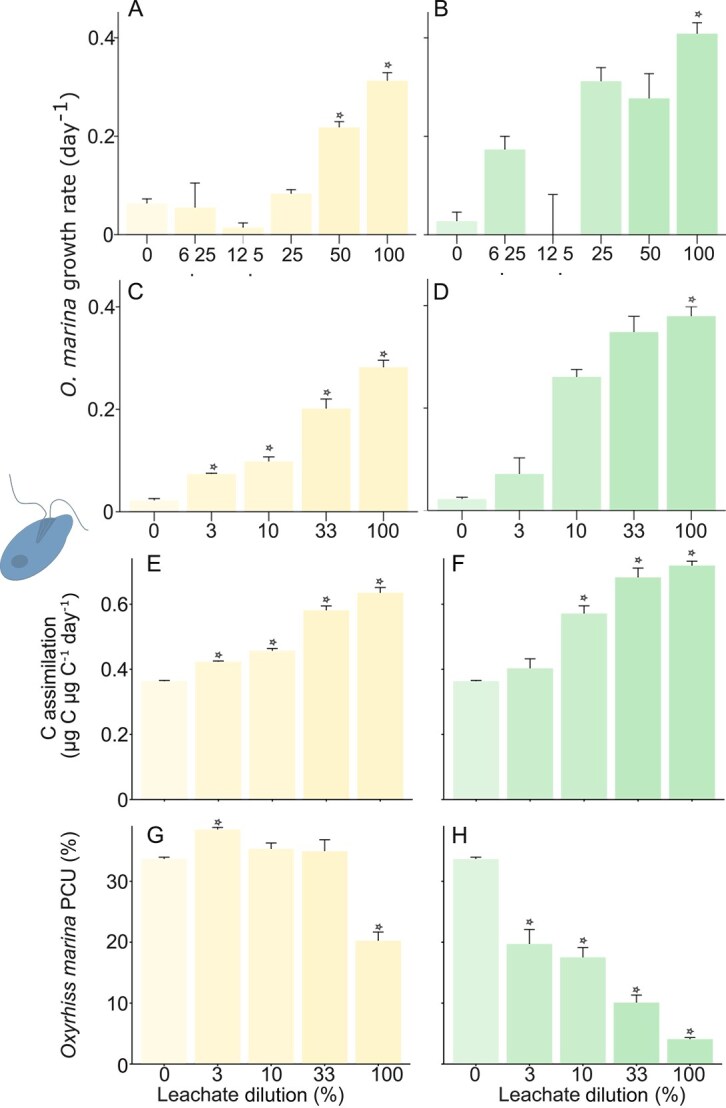
Growth dynamics of *O. marina* in response to leachate from BCP and BioP after 72 h of incubation. A–D: standard growth rate of *O. marina* ( d$^{-1}$) as a function of leachate concentration (%). Plots A and B show data for BCP and BioP in Experiment 1, whereas plots C and D show data for BCP and BioP in Experiment 2. E and F: carbon assimilation ($C_{\text{assimilation}}$) of *O. marina* (in $\mu \text{g}\,\textup{C}\,\mu \textup{g}\,{\text{C}_{O.\ marina}}^{-1}\,\text{d}^{-1}$) as a function of leachate concentration (%) for BCP and BioP, respectively. G and H: proportion of carbon used (PCU) by *O. marina* (%) at varying plastic concentrations for BCP and BioP, respectively. Except for plots A and B, all measurements were conducted during the second *O. marina* growth experiment. Values are presented as mean $\pm $ standard error ($n=3$). Significant differences from the control means are indicated with “*” ($\mathit{P} < 0.05$).

Exposure of *O. marina* to plastic leachates resulted in a concentration-dependent increase in growth rate in both experiments (Fig. 3A–D). This dose–response were statistically significant for both plastics and experiments (Experiment1, BioP, $\mathit{F} = 4.3$, $\mathit{P} = 0.02$ and BCP, $\mathit{F} = 3.5$, $\mathit{P} = 0.04$; Experiment2, BioP, $\mathit{F} = 7.0$, $\mathit{P} = 0.01$ and BCP, $\mathit{F} = 362.7$, $\mathit{P} < 0.001$). The maximum growth rates were observed with undiluted leachates, reaching $\sim $0.3 d$^{-1}$ for BCP and 0.4 d$^{-1}$ for BioP (i.e. almost 2.5 and over three times the initial population for BCP and BioP, respectively, over 72 h). Statistically significant growth increases, compared with the control, were found in *O. marina* exposed to BCP at the highest tested concentrations (50% and 100%; $\mathit{P} = 6 \times 10^{-6}$, $\mathrm{df} = 5$, $\mathit{F} = 24.7$ in Experiment 1, and 33% and 100%; $\mathit{P} = 0.009$, $\mathrm{df} = 4$, $\chi ^{2} = 13.5$ in Experiment 2). Similarly, experiments with BioP leachates showed significant differences in growth compared with the control at the highest concentration (100%; $\mathit{P} = 0.014$, $\mathrm{df} = 5$, $\chi ^{2} = 14.3$ for Experiment 1, and $\mathit{P} = 0.014$, $\mathrm{df} = 4$, $\chi ^{2} = 12.4$ for Experiment 2). The comparison between experiments 1 and 2 underscores the consistency of the observed growth rates, particularly at high plastic concentrations, despite differences in methodologies, such as cell concentration and experimental setups (open vs. closed bottles). This consistency across experiments reinforces the robustness of the results and suggests that the impact of plastic leachates on *O. marina* growth is not significantly affected by experimental variations.

In the second experiment, leachate exposure induced few cell size variations, ranging from −2% to 5% compared with the A-FSW control ($13.8 \pm{0.06}\ \mu $m (Table S1, Supplementary Information). A statistically significant difference was found only between the control and the 33% BCP treatment ($\mathit{P} = 3.25e-2$, $\mathrm{df} = 4$, $\chi ^{2} = 10.5$).

The carbon assimilation ($C_{\text{assimilation}}$) was leachate concentration-dependent (Fig. 3E and F), showing a similar pattern as the growth (Fig. 3C and D). This value was around $0.36\pm{0.002}\ \mu \text{g} \textup{C}\ \mu \text{g} {\textup{C}_{O.\ marina}}^{-1}\,\text{d}^{-1}$ in the control whereas it reached almost $0.63\pm{0.02}\ \mu \text{g}\ \textup{C}\ \mu \text{g}\, {\textup{C}_{O.\ marina}}^{-1}\,\text{d}^{-1}$ and $0.72\pm{0.01}\ \mu \text{g}\ \textup{C}\ \mu \text{g}\, {\textup{C}_{O.\ marina}}^{-1}\,\text{d}^{-1}$ for 100% BCP and BioP leachates, respectively. All values differed significantly from the control with 0% plastic leachates excepted for BioP at 3% ($\mathit{P} = 1.56e-8$, $\mathrm{df} = 4$, $\mathit{F} = 127.4$ and $\mathit{P} = 1.06e-6$, $\mathrm{df} = 4$, $\mathit{F} = 53.1$ for BCP and BioP, respectively).

In summary, exposure to plastic leachates significantly increased *O. marina* cell concentration without a reduction in cell size, leading to a notable rise in total protozoan biomass. This enhanced biomass was reflected in the assimilation of carbon in *O. marina*, which reached nearly 3.3$\ \mu $g C for BCP and exceeded 4.8$\ \mu $g C for BioP over the 72-h incubation period. This observation suggests that the DOC from the leachates acts as a significant carbon source that likely stimulates and supports protozoan growth.

The PCU, calculated as the proportion of available DOC assimilated by *O. marina* during incubation, decreased in a concentration-dependent manner, particularly at the highest leachate concentrations, from $33.6\pm 0.3\%$ in control, to $20.3\pm 1.4\%$ and $4.1\pm 0.3\%$ for BEP and BioP, respectively (Fig. 3G and H). For BioP, all leachate concentrations showed statistically significantly different values from the control. In contrast, for BCP, significant differences were observed only at three and 100% concentrations ($\mathit{P} =4.75e-7$, $\mathrm{df} = 4$, $\mathit{F} = 62.9$ for BioP, and $\mathit{P} = 5.22e-6$, $\mathrm{df} = 4$, $\mathit{F} = 37.8$ for BCP). In conclusion, *O. marina* utilized only a fraction of the available DOC, with the PCU dropping in pure BioP leachate. Even at dilutions of 30 mg l$^{-1}$, both leachates provided more than sufficient carbon to support protozoan growth, with PCU values reaching 39% for BCP leachate and 20% for BioP leachate. This highlights the potential for leachates to act as a carbon source in marine environments.

The saturation of growth rates can explain this phenomenon of plateauing observed in *O. marina*, which is often constrained by the prey size and nutritional quality of the available food source [34]. Various biotic and abiotic factors influence maximum growth rates in *O. marina*, including food quality and availability. In conditions approaching food saturation, *O. marina* achieved growth rates ranging from 0.19 to 1.47 d$^{-1}$, depending on the prey type, such as bacteria or microalgae. The growth rate plateaued at prey concentrations above 100 ng C ml$^{-1}$, which aligns with the observed growth rates of 0.3 to 0.4 d$^{-1}$ for *O. marina* exposed to 1000 mg l$^{-1}$ of BCP and BioP leachates, respectively.

Our results suggest that plastic leachates could serve as a medium-quality carbon source by enabling osmotrophy (i.e. feeding through dissolved molecules) and increasing bacterial concentration that serves as prey for *O. marina*. These trophic modes have been described among HDFs [34, 64]. Considering the particular nutritional requirements of *O. marina* such as Vitamin B12-auxotrophy [65], combining these two mechanisms might be required to allow protozoan growth. The use of water-soluble factors in the nutrition of *O. marina* has already been demonstrated in the case of limited food resources not allowing phagotrophy [64]. As described by the author, it needs one of several essential carbon sources to allow its growth. Thus, acetate concentration was directly proportional to the observed growth rate, but authors also identified several organic compounds as ineffective carbon sources (e.g. glycerol, glycogen, starch). Also, comparable conclusions were drawn for nitrogen sources. Furthermore, another article also showed that *O. marina* growth strictly depends on specific ubiquinone precursors that are probably not encountered in the studied plastics [66]. Theoretical estimates show that bacterivory by *O. marina* contributes <0.2g C g C$^{-1}$ under all conditions (see Fig. S1, Supplementary Information), whereas total carbon assimilation reached up to 0.7g C g C$^{-1}$ in pure leachate. This indicates that bacterial ingestion alone cannot account for the observed growth, and that up to 70% of the assimilated carbon likely comes from direct osmotrophy of dissolved compounds in the leachate.

**Figure 4:**
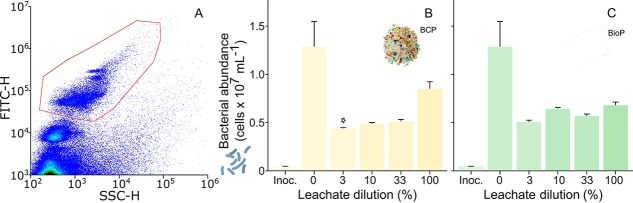
Bacterial response to leachates from conventional plastics (BCP) and BioP after 72 h of incubation. A: identification of heterotrophic bacteria by flow cytometry (example from a control sample, with red polygon representing the gating region). B and C: bacterial abundance (cells ml$^{-1}$) as a function of leachate concentration ($mean \pm standard error$; $\mathit{n} = 3$) for leachates from BCP and BioP, respectively. Significant differences from control means are indicated with “*” ($\mathit{P} < 0.05$). Abbreviation: Inoc. = Inoculum. Measurements were conducted during the second *O. marina* growth experiment.

Bacterial abundance was measured by flow cytometry, where heterotrophic bacteria were identified based on size and fluorescence intensity (Fig. 4A). No bacteria were detected in the control A-FSW or the plastic leachates before adding *O. marina* (data not shown). However, bacteria were present in the *O. marina* culture inoculum, with a mean abundance of $4.75e5 \pm{2.54} \times 10^{3} \,\text{cells}\,\text{ml}^{-1}$ (Fig. 4A).

Following incubation with *O. marina*, bacterial concentrations increased to $1.29e7 \pm{2.61} \times 10^{6} \,\text{cells}\,\text{ml}^{-1}$ in the control. This increase was also observed in the presence of plastic leachates, but it tended to be higher in the control compared with the leachate treatments. A statistically significant difference was observed only when comparing the control with the bacterial concentrations in the BCP leachate diluted at 3% ($\mathit{P} = 1.47e-2$, $\mathrm{df} = 4$, $\chi ^{2} = 12.4$). In the control, the proportion of carbon used for bacterial growth was $\sim $20%, whereas in samples containing leachates, this rate remained below 10%. A clear concentration-dependent decrease was observed in both types of plastic leachates (Fig. 4D and E). Values were statistically significantly different from the control at concentrations ranging from 33% to 100% for BCP ($\mathit{P} = 9.07e-3$, $\mathrm{df} = 4$, $\chi ^{2} = 13.5$) and from 10% to 100% for BioP ($\mathit{P} = 6.14e-6$, $\mathrm{df} = 4$, $\mathit{F} = 382.6$).

The presence of bacteria in the leachate was confirmed by flow cytometry analysis in the *O. marina* culture, with the inoculum promoting bacterial growth across all samples after 72 h of incubation. However, the addition of leachate did not lead to an increase in bacterial concentration, contrasting with the expected bioavailability of leached DOC from both petroleum-based and biodegradable plastics, as reported in other studies [13, 16]. These studies found that bacteria were capable of utilizing over half of the plastic-leached DOC.

The *O. marina* cultures were maintained in darkness, including during the bioassays. Therefore, the associated bacterial community can only be heterotrophic, as confirmed by flow cytometry. Previous studies found that marine heterotrophic bacteria are primarily responsible for the uptake of DOC, including DOC from plastic leachates [16, 61]. *O. marina* is known to form mutualistic or symbiotic relationships with various heterotrophic bacteria. The Alphaproteobacteria from the order Rhodobacterales, including *Oceanicaulis* spp. [67] and *Roseibium aggregatum*, have been reported in association with *O. marina*, with the latter shown to promote its growth [68]. Prior research on the effects of plastic leachates on planktonic microbial communities has identified Rhodobacterales, particularly members of the Roseobacter clade, as a key bacterial group that responds positively to such leachates [69]. Associated bacteria have been observed on the surface of *O. marina* cells even when cultured with antibiotics [67], making it challenging to accurately estimate the contribution of osmotrophy to the total assimilated carbon by *O. marina*.

**Figure 5:**
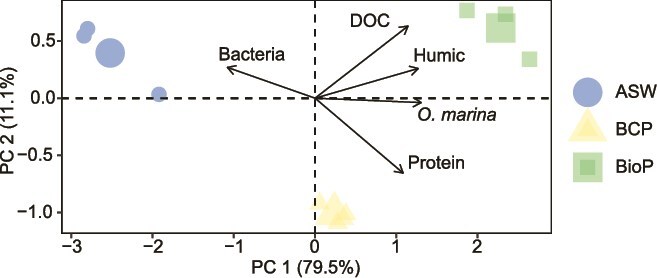
PCA of plastic leachate properties before incubation and *O. marina* samples after 72 h of incubation under different conditions. The larger symbols represent the mean of each condition ($\mathit{n} = 3$). Variables include DOC (concentration of DOC), Protein (integrated fluorescence in the protein-like region), Humic (integrated fluorescence in the humic-like region), Bacteria (bacterial abundance), and *O. marina* (concentration of *O. marina*).

This divergence may be explained by limitations on bacterial concentration in the presence of *O. marina*, potentially due to factors such as grazing pressure or competition for DOC between *O. marina* and the bacterial community. This is further supported by the negative correlation between the bacterial abundance and *O. marina* growth in the PCA representation (Fig. 5). The composition of the bacterial community in the culture could also contribute to the observed results, as not all species exhibit the same capacity to utilize available DOC.

Previous studies have shown that plastic leachates can significantly alter bacterial community composition by stimulating certain copiotrophic or generalist taxa—such as Alteromonadales, Oceanospirillales, Nitrosococcales, and Rhodobacterales—whereas negatively affecting others like SAR11 [70, 71]. Overall, an increase in total bacterial abundance is commonly reported following leachate exposure [13]. However, in the present experiment, bacterial abundance declined at higher leachate concentrations, likely due to a combination of factors. One possibility is increased grazing pressure, as *O. marina* densities rose with leachate levels, potentially intensifying predation on bacteria. Competition for DOC between *O. marina* and bacteria may have also contributed to the decline.

The composition and concentration of plastic-derived DOC are key factors influencing microbial responses. DOC from plastics includes polymer degradation products and additives, which may be stimulatory for some microbes and toxic for others [15, 63]. High concentrations of leachates have been shown to exert toxic effects on microbial communities [13, 72], creating a balance between the harmful influence of certain compounds and the beneficial release of utilizable organic matter. This balance may help explain the mixed bacterial responses observed. Additionally, *O. marina* is typically associated with antibiotic-resistant bacteria that are difficult to eliminate, complicating the establishment of axenic cultures [67]. These associated bacteria, often found attached to *O. marina*, may contribute to its DOC utilization through potential symbiotic interactions. Altogether, the interplay between leachate composition, bacterial community structure, and the physiological traits of *O. marina* likely shapes the complex responses observed in protozoan and bacterial populations under plastic leachate exposure.

The PCA representation (Fig. 5), including the different variables studied in the present experiment, demonstrates that plastic characteristics (DOC, FDOM), bacterial abundance, and *O. marina* cellular concentrations effectively discriminate between the different conditions (i.e. A-FSW and leachates from BCP and BioP). The bacterial concentration generally opposes protozoan concentration across the samples, whereas protozoan abundance is closely associated with the concentration of carbon leached from plastics. These variables likely play significant roles in the observed growth patterns, reinforcing the idea of complex dynamics driving protozoan growth on plastic leachate.

### 3.3 Ecological implications

Even though caution is necessary when extrapolating laboratory results to natural environments, our findings suggest that plastic pollution could significantly impact the marine microbial loop. Specifically, plastic pollution appears to promote the growth of both heterotrophic bacteria and heterotrophic dinoflagellates through the utilization of DOC from plastic leachates. In an era of increasing plastic pollution in the ocean, the relative higher tolerance of some dinoflagellate species to pollutants [36, 73] and the diverse trophic modes of this group could provide an adaptive advantage. The ability of *O. marina* to utilize osmotrophy and bacterivory allows them to survive periods of limited phytoplanktonic prey. Increasing concentrations of these major grazers will elevate grazing pressure on phytoplankton. Globally, stimulating heterotrophic prokaryotes and eukaryotes in microbial communities will increase oceanic respiration, contributing to higher CO$_{2}$ release into the biosphere.

The concentrations of *O. marina* and associated bacteria used in this study are environmentally relevant. Although the wide geographic distribution of this species in coastal waters is well-documented, its field abundance data are limited. Yet, *O. marina* can be found at concentrations of up to 100cells ml$^{-1}$ in summer [74] and can form blooms of up to 4.4 $\times 10^{5}$ cells ml$^{-1}$ in coastal waters [75]. The initial bacterial concentration in our experiment (0.47 $\times 10^{6}$ cells ml$^{-1}$) falls within the range typically found in marine waters (1 $\times 10^{5}$$^{-1}$ to 1 $\times 10^{6}$ cells ml$^{-1}$ ) [46]. Other heterotrophic dinoflagellates and nanoflagellates also exhibit osmotrophy and bacterivory [76] and could respond similarly to *O. marina* when exposed to plastic leachates. Thus, the outcomes observed in this study may be generalized to the marine microbial loop, with different magnitudes depending on the environmental conditions.

Assessing plastic leachate exposure levels in aquatic environments is challenging, as leaching can occur from plastic particles in the water column, during sinking, and on the seafloor and can be affected by multiple environmental factors. The mass of microplastics in the water column is typically lower than used in our experiments, as it is a transient compartment. However, marine plastics can reach high concentrations in surface waters in convergence zones [77] and in coastal areas in the form of marine litter windrows (e.g. 78 kg km$^{-1}$, [78]). Additionally, the mass of plastics on surface sediments in coastal waters can exceed hundreds of mg m$^{-3}$ [79] and the amount of plastic on beaches, exposed to wave and current washing, enhancing plastic fragmentation and leaching, can exceed several g m$^{-3}$ [80].

**Figure 6:**
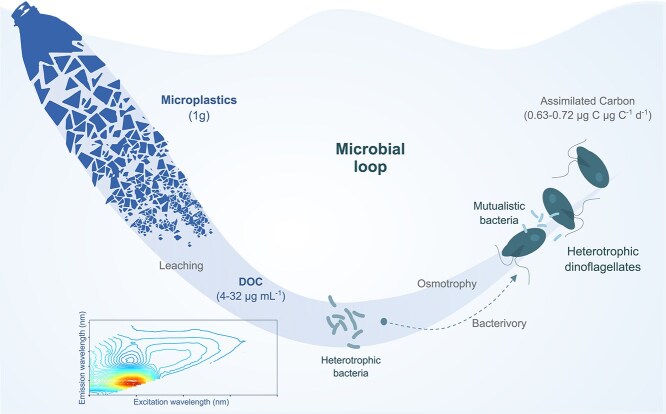
Conceptual diagram of this study. Key processes and ecological implications of our findings on the influence of plastic leachates on marine microbial dynamics are illustrated. The assimilation of plastic-derived DOC into the food web via heterotrophic dinoflagellates and the potential role of their associated bacterial communities is highlighted. The DOC concentrations and carbon assimilation rates presented are based on our experimental conditions; however, their magnitude may vary depending on factors such as the type of plastic, the species of heterotrophic dinoflagellates, and environmental conditions.

Overall, our results indicate that part of the carbon leached by plastics can fuel heterotrophic metabolism by being assimilated into the microbial food web via marine protozoans and bacteria, as indicated in the conceptual model (Fig. 6). *O. marina* can efficiently incorporate in a day up to around 0.7g C g C$^{-1}$ of *O. marina* into the food web per gram of plastic through osmotrophy and bacterivory. In contrast, previous studies show that marine picophytoplankton (e.g. *Prochlorococcus*), major primary producers in the ocean responsible for up to 20% of the oxygen in our biosphere, are negatively affected by plastic leachates. Given the vast amounts of plastic entering marine systems and the contrasting effects of plastic leachates, which are adverse for phytoplankton but promote growth of the heterotrophic microbes observed here, there is a clear need to investigate their potential impact on the global marine production-to-respiration (P/R) ratio and, more broadly, on carbon and oxygen cycling at the planetary scale.

The role of marine plankton communities as either sinks or sources of CO$_{2}$ has been a subject of debate over the past decades [81–83]. The ocean–atmosphere CO$_{2}$ exchange is largely governed by the metabolic balance between photosynthesis by picophytoplankton and microbial respiration [84, 85]. When gross primary production (GPP) roughly equals community respiration (R), resulting in a P/R ratio close to 1.0, the system approaches metabolic equilibrium, as commonly observed in open ocean waters with only minor fluctuations [83]. In coastal environments, however, both net autotrophic (P/R $> 1$) and heterotrophic (P/R $< 1$) conditions have been reported, indicating that these systems can act as either CO$_{2}$ sinks or sources depending on local and seasonal dynamics. The net community production can be influenced by external inputs of organic carbon, particularly in oligotrophic regions where DOC is often limited, such as in the open ocean, stratified surface waters, or during summer months.

Even though our experimental approach focused on *O. marina* as a model organism, it is essential to acknowledge that direct extrapolation to the broader microbial loop should be done with caution. Nevertheless, the metabolic traits displayed by *O. marina*—such as osmotrophy and bacterivory—are shared by a variety of heterotrophic protists in marine ecosystems [76]. The ability of these organisms to exploit plastic-derived DOC may represent a generalizable response among microbial grazers. Results presented here contribute to the growing evidence that plastic leachates can shift microbial community metabolism toward heterotrophy, with potential consequences for the oceanic production-to-respiration (P/R) balance. As such, whereas the specific responses may vary by taxon or environment, the overarching implication—that plastic pollution may enhance heterotrophic processes at the expense of autotrophic ones—remains ecologically significant and warrants further investigation across diverse microbial communities.

 In conclusion, this study provides evidence that plastic leachates can stimulate the growth of marine protozoans, specifically the heterotrophic dinoflagellate *O. marina*, highlighting a previously underexplored impact of plastic pollution on the marine microbial loop. Our results demonstrate that DOC leached from both conventional and BioP significantly enhances the growth rates and biomass of *O. marina*, even in the absence of microalgal prey. This suggests that plastic-derived DOC serves as a viable carbon source for these protists, either through direct assimilation or indirectly via associated bacterial communities.

The findings also underscore that *O. marina* utilizes only a fraction of the available DOC from plastic leachates, yet even at low dilutions, this carbon source is sufficient to support substantial protozoan growth. These results imply that plastic pollution could disrupt marine microbial dynamics by enhancing heterotrophic metabolism, with potential implications for carbon cycling and food web structure. Given the projected increase in plastic pollution, understanding the balance between DOC uptake by heterotrophic organisms and its potential effects on autotrophic processes in the ocean is critical.

Future research should focus on examining the broader ecological consequences of plastic-derived DOC, including potential shifts in microbial community structure and the cascading effects on marine food webs and biogeochemical cycles. Such insights are crucial for developing a more comprehensive understanding of how plastic pollution impacts oceanic ecosystems and the global carbon cycle.

In the context of increasing plastic pollution, including in remote ocean gyres, plastic-derived chemical leachates may exert a dual influence on plankton communities. Whereas DOC released from plastic can stimulate microbial loop activity, enhancing microbial respiration, as observed in previous studies [13] and corroborated here for protozoans. In contrast, toxic additives leached from plastics negatively affect picocyanobacteria such as *Prochlorococcus* and *Synechococcus* [70, 72, 86, 87], major primary producers in the ocean, thereby potentially reducing GPP. Increasing plastic pollution could shift plankton communities toward net heterotrophy, diminishing their capacity to act as CO$_{2}$ sinks and potentially increasing CO$_{2}$ release to the atmosphere. It has been estimated that up to 10% of the DOC in the North Atlantic gyre surface microlayer might come from plastics [13]. Further research is urgently needed to quantify the potential impact of plastic leachates on plankton community metabolism, taking into account the complex interplay of environmental and biological factors that modulate these processes [13, 88].

## Supplementary Material

SI_wraf195

## Data Availability

The raw data used in this study are publicly available at Figshare (https://doi.org/10.6084/m9.figshare.29891216); the code is available from the corresponding author upon request.
