# Supplementary material for: Plastic leachates promote marine protozoan growth
Source: ISME J. 2025 Aug 28;19(1):wraf195. doi: 10.1093/ismejo/wraf195 (PMC12573266; doi:10.1093/ismejo/wraf195)
Supplement: SI_wraf195 [file si_wraf195.pdf]

Supplementary Information for Plastic leachates promote marine protozoan growth

Jessy Le Du-Carrée, Cristina Romera-Castillo, Rodrigo Almeda

August 25, 2025

Supplementary Tables

Table 1: Cell size measurements (mean equivalent spherical diameter, µm) of *O. marina* at the end of the incubation period in the second experiment, showing variations in response to beach conventional plastic (BCP) and bioplastic (BioP) leachates. Values are presented as mean ± standard error ($n = 3$). Significant differences from the control means are indicated with "*" ($P < 0.05$).

Plastic	Dilution	mean	se
BCP	0	13.8	0.1
	3	13.9	0.0
	10	14.0	0.1
	33	14.5*	0.1
	100	14.2	0.1
BioP	0	13.8	0.1
	3	13.6	0.1
	10	13.5	0.3
	33	14.1	0.2
	100	14.4	0.0

Supplementary Figures

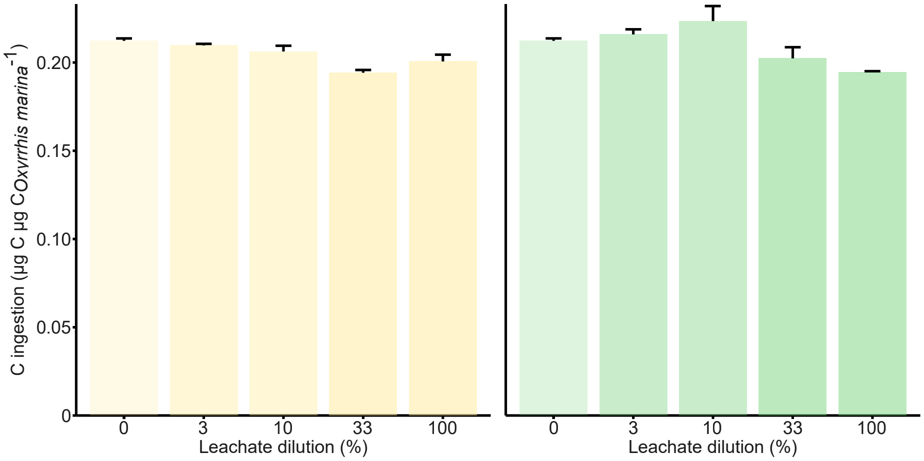

Figure 1: Estimated C-specific ingestion of *O. marina* on bacteria based on experimental data from a previously published study [1] (µg C µg $C_{O.\ marina}{}^{-1}\ d^{-1}$) in response to leachate from beach conventional plastics (BCP, plot A) and bioplastic (BioP, plot B) after 72 h of incubation. Values are presented as mean ± standard error ($n = 3$).

References

[1] Hae Jin Jeong, Kyeong Ah Seong, Yeong Du Yoo, Tae Hoon Kim, Nam Seon Kang, Shin Kim, Jae Yeon Park, Jae Seong Kim, Gwang Hoon Kim, and Jae Yoon Song. Feeding and grazing impact by small marine heterotrophic dinoflagellates on heterotrophic bacteria. *J. Eukaryot. Microbiol.*, 55(4): 271–288, 2008. ISSN 1066-5234. doi:10.1111/j.1550-7408.2008.00336.x.
